# Lung Function and Breathing Pattern in Subjects Developing High Altitude Pulmonary Edema

**DOI:** 10.1371/journal.pone.0041188

**Published:** 2012-07-19

**Authors:** Christian F. Clarenbach, Oliver Senn, Andreas L. Christ, Manuel Fischler, Marco Maggiorini, Konrad E. Bloch

**Affiliations:** 1 Pulmonary Division, University Hospital Zurich, Zurich, Switzerland; 2 Medical Intensive Care Unit, University Hospital Zurich, Zurich, Switzerland; 3 Center for Human Integrative Physiology, University of Zurich, Zurich, Switzerland; University of Adelaide, Australia

## Abstract

**Introduction:**

The purpose of the study was to comprehensively evaluate physiologic changes associated with development of high altitude pulmonary edema (HAPE). We tested whether changes in pulmonary function and breathing pattern would herald clinically overt HAPE at an early stage.

**Methods:**

In 18 mountaineers, spirometry, diffusing capacity, nitrogen washout, nocturnal ventilation and pulse oximetry were recorded at 490 m and during 3 days after rapid ascent to 4559 m. Findings were compared among subjects developing HAPE and those remaining well (controls).

**Results:**

In 8 subjects subsequently developing radiographically documented HAPE at 4559 m, median FVC declined to 82% of low altitude baseline while closing volume increased to 164% of baseline (P<0.05, both instances). In 10 controls, FVC decreased slightly (to 93% baseline, P<0.05) but significantly less than in subjects with HAPE and closing volume remained unchanged. Sniff nasal pressure was reduced in both subjects with and without subsequent HAPE. During nights at 4559 m, mean nocturnal oxygen saturation dropped to lower values while minute ventilation, the number of periodic breathing cycles and heart rate were higher (60%; 8.6 L/min; 97 cycles/h; 94 beats/min, respectively) in subjects subsequently developing HAPE than in controls (73%; 5.1 L/min; 48 cycles/h; 79 beats/min; P<0.05 vs. HAPE, all instances).

**Conclusion:**

The results comprehensively represent the pattern of physiologic alterations that precede overt HAPE. The changes in lung function are consistent with reduced lung compliance and impaired gas exchange. Pronounced nocturnal hypoxemia, ventilatory control instability and sympathetic stimulation are further signs of subsequent overt HAPE.

**Registration:**

ClinicalTrials.gov identifier: NCT00274430

## Introduction

High altitude pulmonary edema (HAPE) is a potentially life-threatening condition that occurs after rapid ascent to high altitude. Mountaineers with a previous history of HAPE are at particular risk. The diagnosis at high altitude is based on the presence of dyspnoea, tachypnea, cough, pulmonary crackles, and cyanosis. A chest radiograph demonstrates pulmonary infiltrates but this is rarely available in the mountains. Since HAPE is preventable and treatable, it would be desirable, if early diagnosis was feasible by detecting physiologic changes that precede overt HAPE. Cremona and colleagues reported on the potential role of closing volume as a sensitive marker of early subclinical HAPE [1]. However, in a previous study we observed increases in closing volume even in some subjects not developing HAPE after rapid ascent to the Capanna Regina Margherita (4559 m) [2]. In contrast, Dehnert and colleagues found no changes in closing volume or other pulmonary function tests in subjects without a history of HAPE ascending to Capanna Regina Margherita [3]. The purpose of the current study was therefore to further investigate physiologic characteristics differentiating subjects developing HAPE from those remaining free of HAPE. While previous studies had separately focused on various specific aspects [1], [3], [4] the purpose of the current study was to perform a comprehensive evaluation including pulmonary function, blood gases and nocturnal polygraphic recordings in subjects developing HAPE in comparison to healthy controls at 4559 m in order to better understand the time course and pattern of physiologic alterations associated with HAPE. We evaluated whether changes in pulmonary function and breathing pattern would herald clinically overt HAPE at an early stage.

## Methods

### Subjects

The study population consisted in 8 HAPE susceptible subjects with a history of previous radiologically documented HAPE and 10 healthy controls without a history of HAPE (controls). Data were collected as part of studies on prevention of HAPE by dexamethasone and tadalafil [5] and on platelet function at altitude [6], respectively. The time period and setting of the study was the same for both groups. Seven of the HAPE susceptible subjects had received placebo and one subject had received the phosphodiesterase inhibitor tadalafil (10 mg twice daily beginning on the day before ascent to altitude) as part of the trial. All other HAPE susceptible subjects included in the prevention trial did not develop HAPE, hence were not included in the present investigation. The subject’s characteristics are summarized in Table 1.

**Table 1:** Subjects characteristics.

	Controls	HAPE-group
Number of subjects (females)	10 (1)	8 (1)
Age (years)	32 (3.5)	40 (9.4)*
Height (m)	1.78 (0.06)	1.76 (0.07)
Weight (kg)	75 (9.0)	74 (9.2)
BMI (kg/m^2^)	23.6 (3.0)	23.8 (2.3)

Values are presented as mean (SD), *P<0.05 vs. controls.

### Study Protocol

Low altitude baseline evaluation including clinical examination, pulmonary function and nocturnal polygraphic monitoring were carried out in Zurich (490 m) within 1 month before ascent to altitude. Control subjects had all examinations with exception of polygraphic monitoring at 490 m. Study participants travelled from Alagna, Italy, 1130 m, to 3200 m by cable car and subsequently hiked 2 hours to the Gnifetti hut (3647 m), where they spent one night. In the following morning, subjects hiked to Capanna Margherita, 4559 m, within 4–5 h and stayed there for 3 days. Daytime examinations were performed 4–6 h after arrival at 4559 m (day 1) and in the mornings of day 2 and 3, nocturnal polygraphic monitoring was performed during all nights at 4559 m. The diagnosis of HAPE was suspected on clinical grounds (excessive dyspnea, tachypnea, dry cough, cyanosis) and confirmed by chest radiography. Subjects with radiographically confirmed HAPE were treated with oxygen and nifedipine and no further tests were performed in them. The last data obtained before the diagnosis of HAPE is reported as endpoint for the current study.

### Measurements

#### Clinical assessment

A medical history was obtained and a physical examination performed. Acute mountain sickness (AMS) was assessed by the Lake Louise consensus scoring system [7]. A score >4 was considered as representing clinically relevant AMS.

#### Lung function and blood gas analysis

Oxygen saturation was measured by pulse oximetry in the sitting position after 15 min of quiet rest. Spirometry and single breath diffusing capacity were performed according to standard techniques (Vmax system, SensorMedics, Fullerton CA, USA) [8], [9]. For diffusing capacity (DLCO), the unadjusted (standard) values and the values adjusted for high altitude (DLCOadj) are reported: DLCOadj = DLCO*(1+0.0031 * [(P_B_ - 47) * 0.21–149]) [8], [9]. Calibrations of the flow meter and gas analyzers were performed several times a day. Reference values were those of the European Community for Steel and Coal [10]. The nitrogen washout test was performed in the same way as in our previous study and described in detail [2]. Maximal inspiratory and sniff nasal pressures were measured according to standard techniques (MicroRPM, Micro Medical, Kent, UK) [11]. Arterial blood gases were measured (ABL 5 Radiometer, Copenhagen, Denmark) and partial pressure of alveolar oxygen was calculated [12].

**Figure 1:**
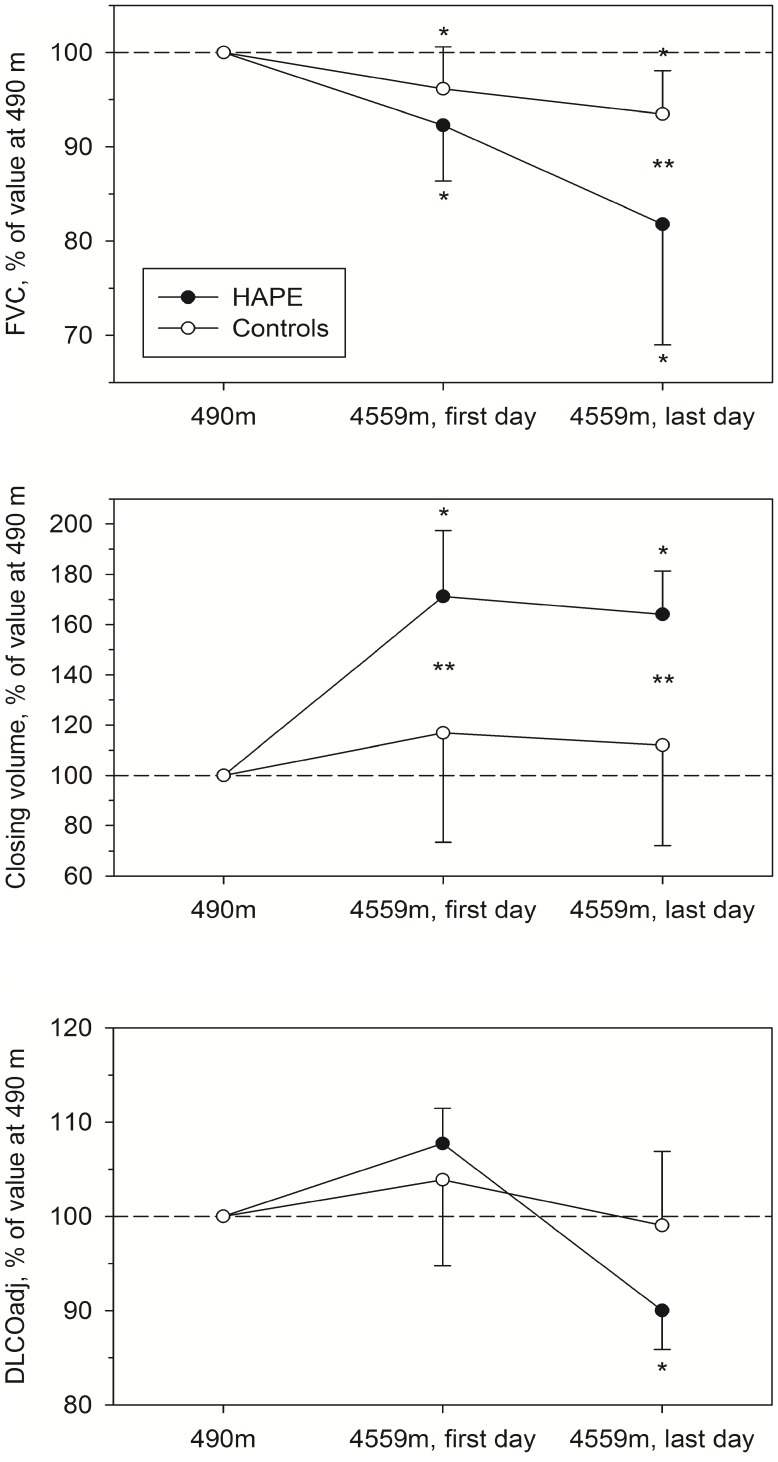
Changes in lung function in subjects developing HAPE and in healthy controls. FVC (upper panel), closing volume above RV (middle panel) and DLCO adjusted for PIO_2_ (lower panel), all expressed in percent of the value at 490 m. HAPE  =  subjects subsequently developing HAPE, controls  =  subjects not developing HAPE; first and last day at 4559 m  =  values measured on the day of arrival and either on the last day before clinically overt HAPE occurred, or on day 3 in controls. *P<0.05 vs. 490 m. **P<0.05 vs. controls.

**Table 2:** Pulmonary function and arterial blood gas analysis.

	Controls (n = 10)			HAPE-group (n = 8)		
	490 m, baseline	4559 m, day 1	4559 m, day 3	490 m, baseline	4559 m, day 1	4559 m, last evaluation
FVC, L	5.64±0.56	5.41±0.48*	5.26±0.53*	5.07±1.32	4.65±1.11*	4.07±0.93*
FVC, % predicted	113±13	109±13*	106±13*	108±17	99±16*	87±15* #
FEV1, L	4.35±0.43	4.28±0.30	4.12±0.46	3.85±1.30	3.60±0.98*	3.23±0.89*
FEV1, % predicted	104±12	102±9	98±1	98±23	93±19	83±18*
FEV1/FVC	77±6	79±7	79±8	75±9	77±6	79±7*
PEF, L/sec	9.38±1.10	10.44±1.29*	10.00±1.13	7.81±2.48	9.05±3.15*	8.61±2.10
PEF, % predicted	99±12	110±15*	105±13	85±20	98±26*	96±23
N_2_ washout, slope of phase III, %N_2_/L	0.83±0.26	0.91±0.32	0.93±0.33	0.83±0.43	1.09±0.62	1.21±0.66*
N_2_ washout, CV, L above RV	0.40±0.10	0.43±0.08	0.41±0.07	0.33±0.06	0.53±0.14* ¶	0.51±0.05* ¶
RV, L	1.52±0.31	1.63±0.42	1.60±0.37	1.63±0.45	1.59±0.37	1.59±0.37
RV, % predicted	85.6±13.2	91.5±19.2	89.9±17.1	84.9±22.8	83.1±18.7	82.8±17.1
TLC, L	6.74±1.17	6.79±0.73	6.79±0.82	6.41±1.43	6.09±1.35	5.48±1.06*
TLC, % predicted	94.9±12.2	97.0±9.9	97.0±9.4	92.6±13.4	88.5±11.7	80.3±10.0*
DLCO, ml/mmHg/min	37.1±6.7	46.4±6.3*	44.6±7.3*	34.1±7.2	45.0±9.0*	37.2±6.2
DLCO, % predicted	107±16	134±17*	129±20*	104±11	138±11*	115±10
DLCO, % pred. PIO_2_ adj.	103±14	107±14	102±16	102±11	109±9	91±8*
Pulse oximetry, SpO_2%_	96±2	79±5*	77±8*	96±1	68±14* ¶	68±11* ¶
SNIP, cmH_2_O	99±23	85±20*	82±27*	70±13	78±14	64±15*
arterial pH	7.41±0.02	7.46±0.03	7.46±0.02*	7.41±0.01	7.48±0.02	7.49±0.01* ¶
PaCO_2_, mmHg	40.2±2.6	30.3±2.1	30.3±1.7*	40.1±2.3	29.0±2.6	28.8±2.0*
PaO_2_, mmHg	89.3±7.3	42.4±2.9*	46.1±2.9*	89.0±3.6	33.4±4.9* ¶	30.1±3.6* ¶
AaPO_2,_ mmHg	14.4±7.5	10.9±3.9	7.5±3.3*	14.7±10.4	21.2±3.5	25.3±2.8* ¶
SaO_2_ (%)	96.5±1.0	80.9±2.5*	83.2±2.9*	96.5±1.3	69.1±8.6* ¶	63.0±7.4* ¶

Means ±SD. FVC, FEV1, PEF: forced expiratory vital capacity, expiratory volume in 1 sec and peak expiratory flow, respectively; CV: closing volume above residual volume. TLC, RV: total lung capacity and residual volume measured by methane dilution; SNIP: sniff nasal inspiratory pressure; DLCO: carbon monoxide single-breath diffusing capacity in absolute units, in % predicted and in % predicted after adjustment for reduced PIO_2_.

* P<0.05 vs. 490 m within group.

¶ P<0.05 vs. controls.

# P = 0.05 vs. controls.

#### Nocturnal polygraphic recordings

Nocturnal polygraphic monitoring was performed and analyzed as previously described [13]. Briefly, a portable device incorporating calibrated respiratory inductance plethysmography, pulse oximetry, ECG and a body position sensor were employed (LifeShirt System, VivoMetrics, Ventura, CA, USA). Inductance sensors were embedded into a snugly fitting shirt. The respiratory inductance plethysmograph was calibrated by the Qualitative Diagnostic Calibration procedure (QDC) during natural breathing [14] followed by fixed volume calibration during rebreathing into a bag of 0.8L for 5 to 10 breaths with the nose clipped. Calibration was verified in the morning after sleep studies and found to be within 20% of the evening value in all instances. Apneas/hypopneas and periodic breathing were scored as previously described. The apnea/hypopnea index was defined as number of apneas and hypopneas per hour. The fraction of the night spent in periodic breathing was measured. The oxygen desaturation index was defined as number of >3% dips per hour.

#### Chest radiography

A chest radiograph was obtained at 490 m and on day 1 and 3 at high altitude or if HAPE was suspected. Chest radiographs were separately scored according to the work by Vock et al. at the end of the study by investigators blinded to the clinical findings and chronological sequence of the examinations [15].

#### Ethics

The ethics committee of the university hospital of Zurich approved the protocol and all subjects gave written informed consent.

#### Data analysis and statistics

Results are shown as means (SD) or medians (quartiles), as appropriate. Measurements at different altitudes and between groups were compared by analysis of variance for repeated measures with Newman-Keuls post-hoc test if appropriate. A probability of P<0.05 was considered significant.

## Results

Eight subjects developed HAPE, 4 on day 2 and 4 on day 3. As per definition, all 8 subjects developing HAPE had radiological and physical evidence of HAPE. Conversely, none of the control subjects had symptoms or clinical signs of HAPE. AMS (Lake Louise score >4) occurred in 7 of 8 subjects developing HAPE (mean score 7.0±2.8) and in 5 of the 10 control subjects (mean score 4.5±2.1, P = 0.04). The tables present mean data (±SD) from subjects developing HAPE and controls for measurements at low altitude (490 m), after arrival at 4559 m and at the time of the last measurement before overt HAPE. The median time lag (quartile range) between the last pulmonary function test and diagnosis of HAPE was 8.5 (4.5–12.0) hours. None of the control subjects developed HAPE. Therefore, their last evaluation was on the third day at 4559 m.

### Lung Function

Results of pulmonary function tests are summarized in table 2 and illustrated in figure 1. On the day of arrival at 4559 m, FVC (% pred.) had fallen significantly by a mean of 4% compared to low altitude baseline in the control group and by 9% in the subjects developing HAPE. There was a further decrease by a mean of 3% in controls and 12% in subjects developing HAPE at their last measurement at 4559 m. FEV1 (% pred.) did not change at high altitude in the control group, however in subjects developing HAPE it was decreased on the last assessment before HAPE occurred. We observed a significant uniform increase in closing volume in the HAPE group at 4559 m, which was in contrast to corresponding values in the control group which revealed variable changes and remained statistically unchanged at altitude compared to 490 m. Moreover the slope of phase III of the nitrogen washout test was increased in the final measurement in subjects developing HAPE. Sniff nasal inspiratory pressure (SNIP) decreased in controls already on the day of arrival at 4559 m while subjects developing HAPE revealed a significant decrease in sniff nasal pressure on their last assessment.

Pulse oximetry revealed lower values in subjects developing HAPE already on the day of arrival at 4559 m and their oxygen saturation remained significantly lower until the end of the study. Carbon monoxide diffusing capacity increased at altitude due to the reduced barometric pressure (and PIO_2_). Adjustment of DLCO for the lower PIO_2_ at altitude revealed no changes in controls but a reduction in subjects developing HAPE in the final measurement.

### Nocturnal Polygraphic Recordings

The results of nocturnal polygraphic recordings are summarized in table 3. In agreement with the daytime measurements, mean oxygen saturation was significantly lower in subjects developing HAPE than in controls already during the first night at 4559 m (62±8% vs. 73±3%, P<0.01). This was in spite of the fact that subjects developing HAPE were breathing faster and at a higher tidal volume than controls and therefore achieved higher minute ventilation. Both groups had pronounced periodic breathing at 4559 m and the highest apnea/hypopnea index was observed in the group developing HAPE.

**Table 3:** Nocturnal polygraphic monitoring.

	Controls (n = 10)		HAPE-group (n = 8)		
	4559 m, night 1	4559 m, night 2	490 m, baseline	4559 m, night 1	4559 m, last night
Recording time, min	472±12	437±65	446±20	438±89	367±138
Mean nocturnal SpO2, %	73±3	73±3	95±1	62±8* ¶	60±8* ¶
Breath rate, 1/min	19.7±2.8	20.0±2.6	17.2±1.4	25.8±5.6* ¶	27.0±4.8* ¶
Tidal volume, L	0.28±0.06	0.25±0.06	0.22±0.07	0.30±0.13	0.32±0.14
Ventilation, L/min	5.6±1.4	5.1±1.8	3.8±1.4	7.6±1.7* ¶	8.6±3.1* ¶
Mean inspiratory flow, L/sec	0.21±0.05	0.19±0.06	0.16±0.05	0.29±0.08* ¶	0.33±0.01* ¶
%Time in Periodic Breathing	35±30	30±29	0±0	56±20*	52±20*
Apnea/hypopnea index, 1/h	58±50	48±44	2±2	101±40*	97±45* ¶
Oxygen desaturation index, dips >3%/h	37±29	35±32	1±1	66±34*	59±35*
Heart rate, 1/min	81±10	79±10	59±7	90±11*	94±9* ¶

Means±SD; 490 m refers to the baseline examinations in Zurich performed in HAPE susceptibles exclusively; night 1 corresponds to the 1^st^ measurement, last night to the last measurement before overt HAPE occurred in the HAPE group and to the measurement in the 2^nd^ night at 4559 m in controls respectively.

* P<0.05 vs. 490 m within group.

¶ P<0.05 vs. controls.

## Discussion

The current study provides a detailed account of respiratory physiologic alterations preceding HAPE in susceptible subjects ascending rapidly to 4559 m. The main changes comprised an initial excessive drop in arterial oxygen saturation associated with a reduction in static lung volumes including closing volume, weakness of inspiratory muscles and an impaired diffusing capacity. Increases in nocturnal breath rate and tidal volume both contributed to augmented minute ventilation with frequent oscillations and severe periodic oxygen desaturations. This pattern is consistent with interstitial fluid accumulation preceding overt HAPE impairing pulmonary diffusion by ventilation/perfusion mismatch and reduction of pulmonary gas exchange surface related to early closure of peripheral air spaces.

Previous studies on physiologic alterations associated with the development of HAPE have mainly focused on hemodynamics, biochemical changes and blood gases. Conversely, data on lung function in subjects developing HAPE have been scant and combined evaluations of pulmonary mechanical changes, gas exchange and breathing patterns have not been reported. Our study closes this gap by providing a comprehensive description of the successive physiologic events.

### Pulmonary Mechanics

Several studies described an early reduction in FVC in mountaineers at altitudes of 3800–4559 m [2], [16], [17] and at simulated altitude of 8848 m in a hypobaric chamber [18] and our results corroborate these data. One single study of Dehnert and colleagues is at variance with these otherwise consistent findings across various altitudes and settings and there is no obvious reason for the discrepancy [2], [3].

Compared to the controls, the subjects developing HAPE had a greater fall in FVC at 4559 m in the measurement that preceded overt HAPE. Reductions in inspiratory muscle force have been reported previously in hypobaric and normobaric hypoxia and have been correlated with reductions in FVC [19]. The reasons for the fall in FVC and TLC are most likely a combination of reduced lung compliance due to interstitial fluid accumulation and inspiratory muscle weakness as suggested by the reduced SNIP preceding HAPE (table 2) [1], [2]. A less favorable length-tension relationship at lower lung volumes and other, yet unknown, factors may additionally have impaired the force generating capacity of inspiratory muscles.

An increase in closing volume associated with a reduction in vital capacity has been suggested to reflect subclinical pulmonary fluid accumulation in mountaineers [1]. The trend of alterations in closing volume and FVC at 4559 m we observed in subjects subsequently developing overt HAPE is consistent with these assumptions (figure 1) and the fact that closing volume was increased in each of them before symptomatic HAPE necessitated treatment suggests sensitivity of this variable as a harbinger of overt HAPE. In a previous study at 4559 m [1], closing volume was increased in 34 of 37 mountaineers even without HAPE-related symptoms but with physical diagnostic and/or radiological evidence of (subclinical) HAPE. Premature closure of peripheral air spaces due to a reduction in lung compliance by interstitial fluid accumulation is the most likely explanation for the increased closing volume since similar statistically significant increases in closing volume did not occur in our controls (table 2) in whom closing volume revealed no uniform trend with ascent to and during stay at 4559 m. Differences in ascent rate and the individual level of physical exertion during climbing might have contributed to the variability of closing volumes in subjects without subsequent HAPE since strenuous exercise promotes interstitial pulmonary edema [20]. An increased closing volume is therefore not a specific marker of HAPE. Since spirometry did not show any evidence of airflow obstruction we do not assume that the increase in closing volume can be explained by exercise or cold air-induced bronchial constriction.

### Pulmonary Gas Exchange

We observed that diffusing capacity (adjusted for the reduced PIO_2_) remained unchanged in controls during the stay at 4559 m while subjects developing HAPE revealed a decrease in DLCO before overt HAPE occurred. In subjects developing HAPE, DLCO fell by more than 10% below low altitude baseline (figure 1) suggesting impairment in the membrane component of diffusion due to interstitial edema although simultaneous changes in pulmonary capillary blood volume cannot be excluded [21]. Pulmonary gas exchange may also have been impaired by uneven distribution of ventilation as suggested by the increased slope of phase III in the nitrogen washout test (table 2) with consecutive ventilation/perfusion mismatch. Furthermore, the premature peripheral airway closure commented above might have affected diffusion by reducing the gas exchange surface of the lung. In line with the reduced diffusing capacity, the arterial oxygen saturation was excessively decreased during the day and the night in subjects developing HAPE and the alveolar-arterial PO_2_ gradient was increased (table 2).

### Nocturnal Polygraphic Recordings

Consistent with an impaired pulmonary gas exchange and reduced arterial oxygen saturation after arrival at 4559 m, subjects subsequently developing HAPE also revealed a reduced nocturnal oxygen saturation already during their first night at altitude. Furthermore, they increased their ventilation, mean inspiratory flow and heart rate consistent with an increased respiratory center drive and a high sympathetic tone induced by progressive hypoxemia and interstitial edema stimulating pulmonary stretch receptors (table 3). Since high altitude periodic breathing is triggered by hypoxia and increased ventilation, this finding might explain the higher frequency of apneas/hypopneas in the last recordings preceding HAPE in susceptible subjects. Whether increases in pulmonary artery and pulmonary capillary pressure may have contributed to periodic breathing through stimulation of strain receptors similar to the findings in patients with pulmonary hypertension of other etiology [22] or in patients with left heart failure [23] remains open.

In conclusion, our data demonstrate that clinically overt HAPE is preceded by a pattern of alterations in pulmonary mechanics and gas exchange that are consistent with reduced compliance and respiratory muscle strength, uneven distribution of ventilation and gas exchange impairments that are caused by interstitial fluid accumulation. Future studies might evaluate simple, portable measurement techniques suitable for screening mountaineers in the field for early evidence of a physiologic pattern indicating pulmonary interstitial fluid accumulation as an aid in the prevention of overt HAPE.
